# Development of a complex arts-based intervention for patients with end-stage kidney disease whilst receiving haemodialysis

**DOI:** 10.1186/s40814-021-00868-2

**Published:** 2021-06-16

**Authors:** Claire Elizabeth Carswell, Joanne Reid, Ian Walsh, William Johnston, Jenny B. Lee, Helen McAneney, Robert Mullan, Hugh Nelson, Michael Matthews, Elizabeth Weatherup, Andrea Spencer, Jean Michelo, Anne Quail, Grainne Kielty, Alistair Mackenzie, Jenny Elliott, Helen Noble

**Affiliations:** 1 School of Nursing and Midwifery, Queen’s University Belfast, Belfast, UK; 2 School of Medicine, Dentistry and Biomedical Sciences, Queen’s University Belfast, Belfast, UK; 3 Kidney Care UK, Alton, UK; 4 College of the Arts, Center for Arts in Medicine, University of Florida, Gainesville, USA; 5 Centre for Public Health, School of Medicine, Dentistry and Biomedical Sciences, Queen’s University Belfast, Belfast, UK; 6 Antrim Area Hospital, Northern Health and Social Care Trust, Antrim, UK; 7 Northern Health and Social Care Trust, Antrim, UK; 8 Arts Care Northern Ireland, Belfast, UK; 9 Northern Ireland Kidney Patient Association, Belfast, UK; 10 Renal Unit, South Eastern Health and Social Care Trust, Belfast, UK

**Keywords:** Haemodialysis, Intervention development, End-stage kidney disease, Chronic disease, Arts-based intervention, Arts in health

## Abstract

**Background:**

Patients with end-stage kidney disease who receive haemodialysis experience a protracted treatment regimen that can result in an increased risk of depression and anxiety. Arts-based interventions could address this unique issue; however, no arts-based interventions have been developed for delivery within a haemodialysis unit and evaluation within a randomised controlled trials (RCTs).

**Aim:**

To develop a complex arts-based intervention for patients with end-stage kidney disease whilst receiving haemodialysis.

**Methods:**

The development process utilised the Arts in Health framework (Fancourt, 2017). The framework was addressed through the establishment of an interdisciplinary advisory group, collaboration and consultation with stakeholders, a scoping and realist review, shadowing of artists-in-residence, personal arts practice and logic modelling.

**Results:**

The intervention involved six 1-h long, one-to-one facilitated sessions focused on creative writing and visual art. Patients could choose between art form and self-select a subject matter. The sessions had a primary focus on skill development and were delivered using principles derived from the psychological theory of flow.

**Conclusion:**

The Arts in Health framework provided an appropriate and pragmatic approach to intervention development. Complex arts-based interventions can be developed for the purpose of evaluation within a trial framework. This intervention was designed to strike a balance between standardised components, and a person-centred approach necessary to address existential boredom.

**Supplementary Information:**

The online version contains supplementary material available at 10.1186/s40814-021-00868-2.

## Key messages regarding feasibility

- What uncertainties existed regarding the feasibility?

Patients receiving haemodialysis experience high rates of mental health issues, in part as a result of the treatment burden they experience. Arts-based interventions are well placed to address this burden; however, due to their person-centred, flexible delivery they are rarely developed using a robust intervention development framework, or evaluated using trial methodology.

- What are the key feasibility findings?

Using a development framework designed for arts-based intervention, a complex arts-based intervention was developed that was underpinned by both existing theory and evidence, whilst also being guided by patients, healthcare professionals, artists and researchers.

- What are the implications of the feasibility findings for the design of the main study?

The framework and approach to intervention development promoted acceptability and delivery within both a highly specialised clinical setting and trial framework. The subsequent feasibility study [1] demonstrated that evaluation of this intervention is feasible within a definitive trial.

## Background

Chronic kidney disease (CKD) is a progressive disease that is characterised by the gradual loss of kidney function, eventually progressing to kidney failure, also known as end-stage kidney disease (ESKD). ESKD is associated with an intensive treatment regimen that can include multiple medications, dietary restrictions, fluid restrictions and renal-replacement therapy (RRT) [2]. The most common form of RRT is kidney transplantation; however, for patients who are unable to match with a donor or are not eligible to undergo transplantation because of age, frailty or co-morbidities [3], the most common form of RRT is haemodialysis [2].

Haemodialysis is a time-consuming treatment, as it requires patients to attend hospital three times a week for up to 4 h each time. During treatment, the patient is confined to a bed and is connected to a dialysing unit that filters their blood and removes waste products and excess fluid, replacing the role of the kidneys. Whilst haemodialysis is associated with difficult physical symptoms caused by the build-up of fluid in the body between sessions, electrolyte imbalances and the accumulation of waste products [4, 5], some of the most profound issues are associated with the impact of the practicalities of haemodialysis treatment and how this effects patients psychologically, socially and existentially [6].

Time on haemodialysis is a concerning issue for patients receiving haemodialysis [7]. Whilst the time allocated to haemodialysis can impact on a person’s ability to retain employment and sustain a social life [8], the experience of being restricted to a bed or chair for 4 h, three times a week, is in itself a distressing aspect of treatment. Qualitative research has elucidated that the perception of time during haemodialysis is warped, with patients perceiving time as ‘dragging’ as they watch the clock, due to lack of opportunity to engage in meaningful activities [9]. This experience of empty time has been linked to mental health issues, such as anxiety and depression, due to the experience of existential boredom [10]. Existential boredom is a philosophical term used to describe the experience of indefinite waiting, causing a person to dwell on existential issues such as illness and mortality, resulting in intrusive negative thoughts, worry and rumination [9]. The estimated prevalence of depression and anxiety for patients receiving haemodialysis typically ranges between 20 and 40% [11, 12], although rates as high as 78.5% have been recorded when account for a range of depressive disorders [13]. Depression is associated with decreased treatment adherence and quality of life in patients receiving haemodialysis [14], as well as increased symptom burden, morbidity and mortality [15]. Yet, depression remains under-diagnosed and under-treated [11].

### Arts on dialysis

Qualitative research exploring patients experiences of haemodialysis has highlighted the arts as a potential avenue for addressing the issue of empty time and improving quality of life and mental health [9]. Arts-in-medicine programmes have been utilised in practice, both in the UK and internationally, to provide patients receiving haemodialysis with meaningful activity during treatment [16]. Published evaluations of these programmes have identified the experience of time passing quickly as a core benefit [17],whilst qualitative explorations of these programmes have found that whilst patients engage in the arts they are not attending to distressing thoughts [18]. Therefore, the arts demonstrate potential to address the issue of both time and existential boredom for patients receiving haemodialysis.

However, there is a lack of evidence assessing the effect of these interventions on patient outcomes using rigorous quantitative methodological frameworks such as randomised controlled trials (RCTs). This has practical implications for obtaining and sustaining funding for arts-in-medicine programmes in healthcare settings, due to the perception of RCTs as the ‘gold standard’ in research and the emphasis healthcare policy places on empirical evidence when recommending interventions [19]. Therefore, an intervention must be developed that can be delivered within a trial framework to ensure the evidence base can be translated into policy. The aim of this paper is to describe the development process of a complex arts-based intervention that was designed for delivery within pilot cluster RCT [20].

## Methods

### Aim

Development of a complex arts-based intervention for patients receiving haemodialysis, to improve quality of life and mental health, and that could be delivered within a pilot cluster RCT [20].

### Design

A combination of approaches was used to address the components of the intervention development framework. The main approaches were target population based, evidence and theory based and intervention-specific based [21].

### Characteristics of intervention participants

Patients who have end-stage kidney disease and are receiving haemodialysis, who are over the age of 18 and who are able and willing to participate.

### Purpose of intervention

- Address the issue of empty time whilst on haemodialysis and improve quality of life and mental health.
- To address the existing gap of RCTs in the evidence base. The intervention was developed to be delivered within a pilot cluster RCT as part of a larger feasibility study. The development process focused on enabling replication within an RCT whilst containing components thought to trigger mechanisms that could reduce existential boredom. The protocol for the feasibility study, including details on evaluating the intervention, has been published separately [20].

### Development framework

**Fig. 1:**
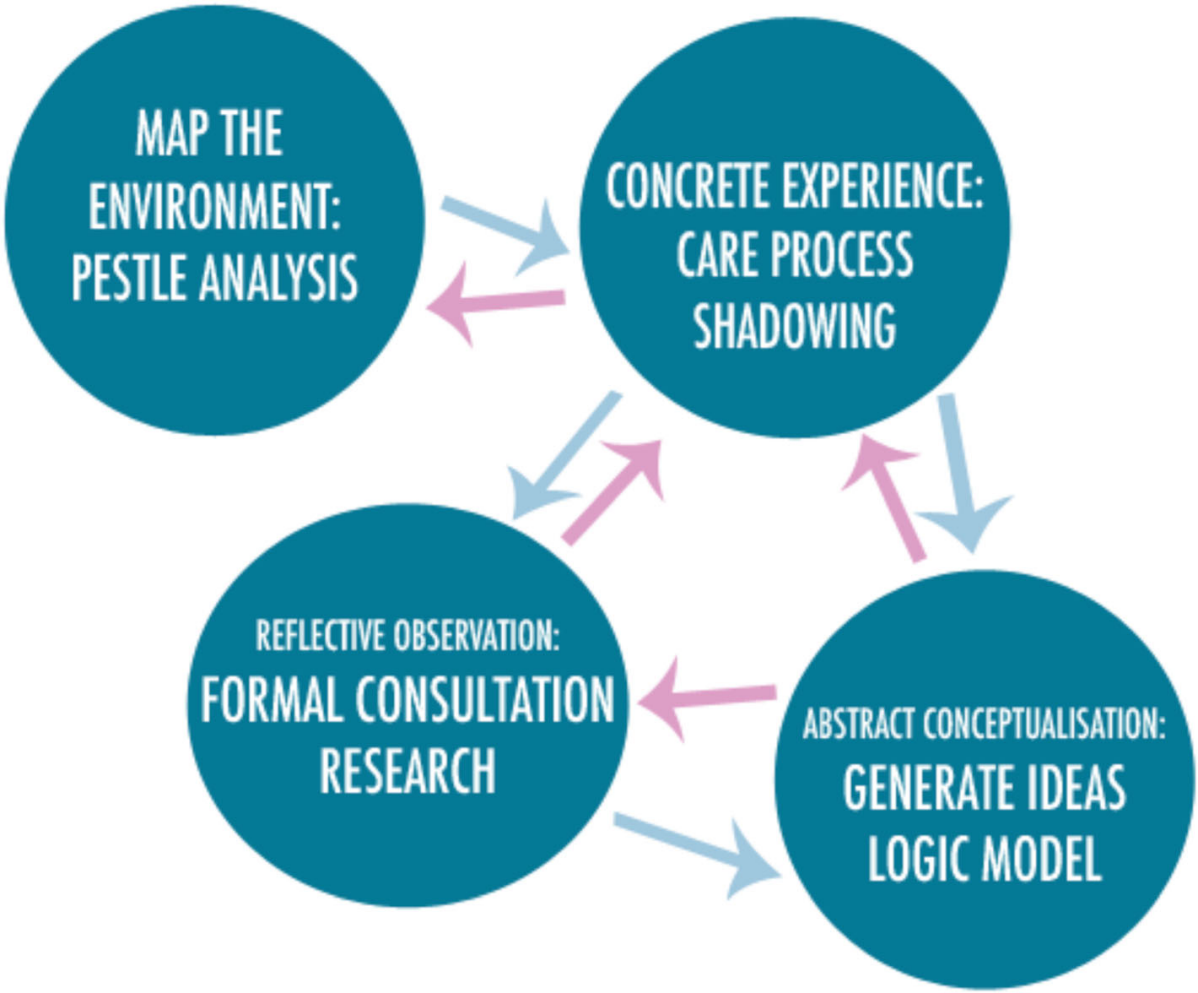
First four steps of the Arts in Health framework for intervention development

The Arts in Health framework [22] guided the development process. The framework consists of seven iterative and flexible steps that cover the process of development, implementation and evaluation. Figure 1 illustrates the steps of the framework that were used to develop the intervention for this study. The final three steps of the framework relate to the feasibility study, the definitive evaluation of the intervention and its efficacy, and long-term implementation in practice; therefore, these were not explored as they are outside the scope of this article. The first four steps of the framework relate specifically to the initial development of the intervention prior to the feasibility study, and will be covered in this paper. These steps include mapping the environment, gaining concrete experience, conducting reflective observation and undertaking abstract conceptualisation. These steps are not intended to be linear and prescriptive but instead provide a framework of considerations that can help ensure the arts-based intervention meets the needs of patients and is feasible to implement in the clinical context [22].

These four steps were considered continuously throughout the development process that lasted from the commencement of the study in September 2017 to delivery of the intervention within the pilot cluster RCT in October 2018 [20], as the process is not sequential and is designed to be iterative and flexible. The information gathered during interdisciplinary advisory group (IAG) meetings, discussions with healthcare professionals, academics and artists, the scoping review and the realist synthesis [16] were reflected upon to identify the core components that needed to be considered for each step of the framework.

The following components were used to address the four steps of the development process:

#### Patient and public involvement (PPI)

The research priorities and questions were identified and set by patients who had lived experience of haemodialysis treatment. The intervention development process primarily utilised the consultation and collaboration approaches to PPI outlined by INVOLVE [23], by placing patients, healthcare professionals and other stakeholders as key contributors to the research process, as opposed to research participants. Patients and other key stakeholders, such as healthcare professionals, were core members of the IAG and contributed to the intervention development, design of the feasibility study and dissemination of findings. In addition, patients and healthcare professionals, separate to the IAG, were consulted during the development process. Kidney patient advocacy groups also provided guidance and support throughout the development process, the conduct and dissemination of the research.

#### Establish interdisciplinary advisory group (IAG)

The IAG included a consultant Nephrologist and research nurse from the renal unit where the intervention would be delivered, three artist representatives from Arts Care Northern Ireland, the CEO of Arts Care Northern Ireland, three patient representatives from the Northern Ireland Kidney Patient Association, members of the Renal Arts Group at Queen’s University Belfast, a representative from University of Florida Center for Arts in Medicine, two managers of Community Well-being and the Renal Counsellor from the Northern Trust, Northern Ireland, a renal social worker from the South Eastern Trust, Northern Ireland, and a statistician. Members of the IAG were selected due to their experience with end-stage kidney disease, either lived, professional or academic, or their experience with arts in health projects. Each member was invited to join the IAG over e-mail or during face to face meetings and an additional advertisement was posted on the Northern Ireland Kidney Patient Association’s Facebook page inviting interested members to join. Prior to delivery of the intervention, there were two formal meetings of the IAG, as well as numerous individual meetings with members when timely advice was needed on aspects of delivery or evaluation.

#### Review existing evidence base

##### Scoping review

The aim of the scoping review was to explore the existing evidence base for arts-based interventions implemented in haemodialysis units, and identify core components of existing arts-based practices that could be translated into a replicable arts-based intervention.

A search of electronic databases was conducted; the databases searched included Cochrane Database of Systematic Reviews, Medline, PsycINFO, Embase, CINAHL, Web of Science and Scopus; these were searched from their conception to January, 2018. The initial search strategy included search terms related to ESKD and haemodialysis, such as ‘Kidney Diseases’, ‘Kidney failure’ and ‘Renal Dialysis’, combined with search terms related to arts-based interventions such as ‘Art’, ‘Music’, ‘Dancing’ and ‘Drama’. Articles were included if they described the delivery or implementation of an arts-based intervention within a haemodialysis unit, and were excluded if they did not describe an empirical study, or if the intervention met the definition of ‘art therapy’. We excluded ‘art therapy’ as this is theoretically, practically and professionally distinct from arts-based interventions. Art therapy is a form of psychotherapy, provided by registered art therapists and is underpinned by distinct theoretical frameworks based in psychotherapy [15].

Due to the dearth of evidence available on arts-based interventions for patients receiving haemodialysis, an additional search was conducted exploring the use of arts-based interventions for patients with cancer [24]. Articles were included to supplement the gaps in the evidence base for patients receiving haemodialysis, specifically if they described the delivery of an arts-based intervention within a clinical setting, and were excluded if they involved the delivery of a music-listening intervention due to their over-representation in the haemodialysis literature. The abstracts and articles were screened by CC, HN, IW and JR, who also developed and revised key themes relating to the review questions.

The findings of the scoping review were discussed with the IAG during a formal meeting on the 26th of June 2018 to ensure members felt the identified components were appropriate, and discuss issues raised within the literature.

##### Realist synthesis

A realist synthesis was conducted that identified aspects of context, mechanism and outcome that needed to be considered during development of a complex arts-based intervention for patients receiving haemodialysis [16]. This review utilised similar search terms as the scoping review for patients receiving haemodialysis, but different inclusion criteria were to ensure only complex interventions were included and additional articles, such as editorials and theoretical articles, were included to inform candidate theories and the development of a theoretical framework to guide the intervention.

#### Personal arts practice

Throughout the development process the primary researcher (CC) maintained a personal arts practice, as advised by the IAG [25].

#### Shadow artists in residence

Engagement with Arts Care also allowed the primary researcher (CC) opportunity to shadow artists in residence to observe the implementation of art within a clinical setting, including a haemodialysis unit at Belfast City Hospital, on the 6th of June 2018, and the 10th of August 2018.

#### Consultations with patients and healthcare professionals

Consultations on the experience of haemodialysis and feedback on components for the arts-based intervention were conducted with patients and healthcare professionals during visits to three haemodialysis units throughout Northern Ireland and attendance at Northern Ireland Kidney Patient Association Meetings.

#### Develop logic models

Logic models are tools that are used to graphically depict the inputs, processes and outcomes of an intervention [26], in a way that allows consideration of the resources available, assumptions underlying successful implementation, consideration of potential barriers and theoretical frameworks guiding each stage of the process [27].

#### Pilot and feasibility testing

This intervention was developed with the purpose of undergoing rigorous evaluation within an RCT, and therefore a feasibility study with an inbuilt pilot cluster RCT was necessary following development. This article outlines the development of the intervention, prior to the feasibility study [20], which was registered at Clinicaltrials.gov (NCT03629496).

## Results

### Step 1: map the environment

The first step of the development process is mapping the environment in which the intervention will be delivered. The framework [22] recommends undertaking a PESTLE analysis to acquire an overview of external factors that may determine whether the environment, either macro or micro, will be supportive of an arts-in-health intervention [28]. A PESTLE analysis outlines that political, economic, social, technological, legal and environmental factors should be considered. The information presented here was obtained from reviewing the literature, IAG meetings, site visits and consultations with patients, healthcare professionals and key academics in the field. This mapping was in anticipation of delivering an arts-based intervention within a pilot cluster RCT conducted in Northern Ireland; therefore, this map will be revised prior to a definitive evaluation, and is not generalisable outside the immediate context of this study.

#### Political

The current political climate of the UK is becoming more supportive of the use of arts in the healthcare context. The All-Party Parliamentary Group for Creative Health and Well-being in the UK recently published a report advocating for increased use of arts in healthcare settings and outlined the potential benefits the arts may have to both patients and the National Health Service (NHS) [29]. Additionally, the Arts in Health Early Career Research Network was established in 2017 to link arts and health researchers, facilitate access to resources and promote future research. This is a global network with representatives in numerous countries, illustrating the international recognition of this growing field. The Center for Arts in Medicine at the University of Florida has been at the forefront of developing the field as a professional practice, providing training and education for practitioners and improving the evidence base through research [30].

#### Economic

The overall economic environment within arts-in-health is precarious in the UK due to limited resources in the NHS and reliance on voluntary organisations for implementation. Consequently, there is increased demand from funders for quantifiable measures of the effects of arts-based interventions. This provides additional impetus for high quality research such as RCTs to ensure that funding remains available. On a micro level funding needed to be acquired to purchase the materials necessary to deliver the arts-based intervention. This was sought through the Northern Ireland Kidney Patient Association and Kidney Care UK, who provided £874.77 for art materials for this study. The long-term sustainability of the intervention, once acceptability and feasibility are determined, will depend on the input from both voluntary organisations and the infrastructure of the NHS Trust in which the intervention is being delivered.

#### Social

The demographics of a targeted group can influence the design of an intervention or approach to implementation, through cultural differences or even the proportion of patients targeted by the intervention. This was discussed within the IAG:

120 patients, majority male. The unit has 4 bays, with approximately 4/4/3/6 beds in each. The process of haemodialysis was described and it was highlighted that a good proportion of time is spent by patients in the foyer prior to commencing haemodialysis. Therefore, interventions do not necessarily need to be intradialytic. The shift patterns were also described; it was explained that patients who attend the evening shift tend to be in better health in comparison to patients who attend the afternoon shift. The importance of considering shift patterns during recruitment was highlighted. (Advisory meeting, 22.2.18)

#### Technological

As a result of the expansion of technology within society, there is potential for this to be used within arts-based interventions. The use of technology within the intervention was discussed with the IAG. The incorporation of digital technology into arts-based activities can also help reduce issues associated with the clinical context, for example using drawing and painting apps to limit the need for messy arts materials [31]; however, the limited resources available within this study meant incorporating digital arts was not feasible.

#### Legal

The most pertinent aspects of law and policy that were considered related to delivery of the intervention within research. The intervention had to be delivered within an ethical framework approved by a research ethics committee, resulting in restrictions around who would be eligible to participate and how participants could be approached. Artists and patient representatives recommended that patients should be actively encouraged to participate and immediately start the sessions to prevent patients from becoming self-conscious. However, due to ethical restrictions of the research project, patients needed an adequate period of time to consider participating in a research study. The legal and ethical restrictions around research meant the recruitment approach used by artists in practice would not be appropriate.

#### Environmental

The intervention was developed with the aim of being delivered during haemodialysis treatment, an outpatient treatment performed in hospital in a specialised clinical setting. The main concerns around delivery related to the context of the clinical environment, including infection control protocols, disruption of the clinical treatment and maintenance of vascular access. Haemodialysis involves two needles being placed into an arteriovenous fistula (a surgically created pathway between an artery and a vein), one needle removes the blood and transfers it to the dialysis machine, the other needle returns the filtered blood to the body. This process continues for 4 h and the placement of the needles must be maintained, as dislodgement of the needles is life threatening [32]. Therefore, the materials and activities had to be tailored to ensure ease of use due to these restrictions.

### Step 2: gain concrete experience

The second step of the development framework involves developing an understanding of the patients’ experience of their health condition [22]. In this circumstance, the focus was on the experience of ESKD, for patients who were receiving haemodialysis. The three patient representatives of the IAG all had experience of in-hospital haemodialysis treatment and highlighted the impact that time on haemodialysis had on their mental health and well-being. All three representatives spoke of the benefits the arts had on their mental health and emphasised the main motivating factor for initial engagement was the desire to fill empty time.

#### Mapping the care process and shadowing the target group

The process of haemodialysis treatment was mapped by the primary researcher (CC) through discussions with the patient and healthcare professional representatives of the IAG, as well as visits to local haemodialysis units to gain an insight into the clinical context and experience of the process. Further understanding of patient experience was gained through attendance at Northern Ireland Kidney Patient Association Meetings and consultations with patients and healthcare professionals during visits to haemodialysis units. The overview of the care process was reviewed and approved by the IAG.

**Fig. 2:**
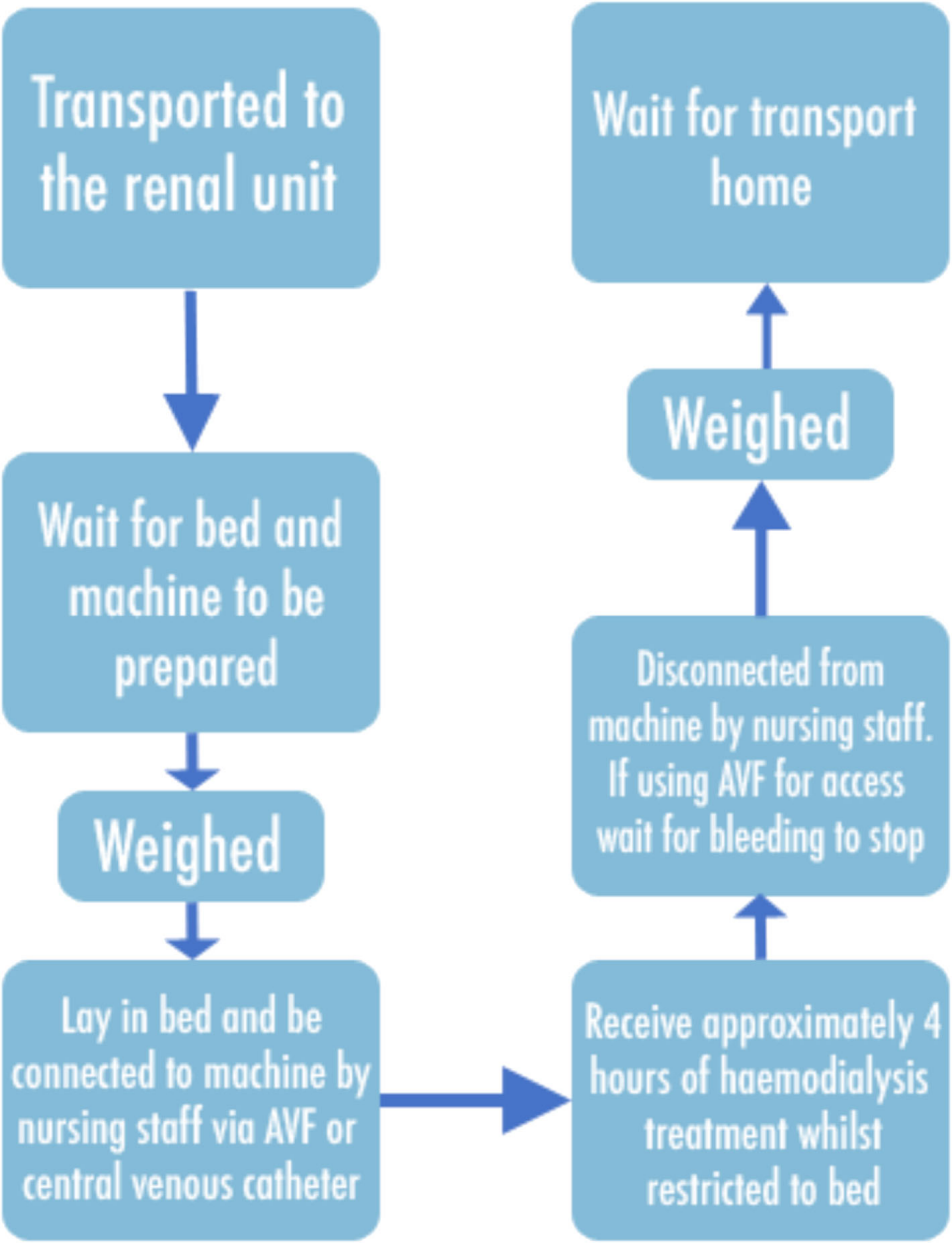
Haemodialysis treatment process

The haemodialysis process involves patients attending hospital three times a week for a period of approximately 4 h each time [32]. Patients are provided transport to and from the hospital by the renal unit; therefore, an additional portion of their day is spent travelling to and from treatment. Once patients arrive, they are seated in a waiting area until their bed and dialysis machine are ready. This wait can be brief or can be prolonged depending on the demands placed on the unit’s resources and healthcare staff. Patients are then weighed and access to their blood supply is achieved either through insertion of two needles into an arteriovenous fistula (AVF), typically placed in the non-dominant arm, or a central venous catheter placed in the chest. An AVF is a form of vascular access that is created through surgical connection of an artery and vein; these results in an enlarged and thickened blood vessel that can withstand repeated access required for haemodialysis. Through this access point, the needles are connected to a hollow tube that delivers their blood to the dialysis machine for filtration [32]. In renal units within Northern Ireland, patients receive haemodialysis whilst supine on a hospital bed, and some patients need to be isolated during treatment due to risk of infection. During treatment, some patients may experience difficult and unpleasant symptoms, such as nausea and cramping, due to changes in fluid levels and electrolytes [4]. Once the treatment is finished, needles are removed or the catheter is disconnected and patients are weighed again. Patients may need to wait for an additional period of time for their transport to arrive before they return home [33]. If there are complications during haemodialysis or following treatment, a patient may be required to wait on the unit until a medic is available to conduct an assessment, which may be several hours depending on the urgency of the complication. If the complication is assessed as requiring closer assessment or treatment, then the patient may be admitted to hospital. This process is illustrated in Fig. 2.

### Step 3: conduct reflective observations

#### Formal consultation

The third step of the framework suggests that a formal consultation should be conducted to ensure identified problems are experienced by the larger target group [22]. There is an established evidence base identifying time and profound boredom on haemodialysis as a significant issue that contributes to poor mental health and quality of life [7, 10], as well as published qualitative research exploring patients experiences of arts-in-medicine programmes [18]. Therefore, consultations with patients and healthcare professionals were conducted to ensure that information and opinions informing the development were representative, in addition to formal meetings of the study’s IAG.

Collaboration with the IAG highlighted the importance of flexibility and a person-centred approach to promote engagement. Initially, to measure adherence and fidelity, it was suggested that the intervention involve a standardised schedule of artistic activities. However, it was highlighted by patients in the IAG that this approach would not appeal to those with a particular interest in a certain art form or material, and would not allow patients the chance to work on larger projects that would require more than a single session to complete.

The importance of a flexible approach and not being prescriptive was emphasised. Participants should be allowed to select the medium that they prefer and follow artistic products through to completion. These mediums should be restricted to what is feasible in the clinical setting and what the researcher feels comfortable facilitating. (Advisory meeting, 22.2.2018)

The consultations with patients provided insight that reinforced the IAG’s opinion that a more person-centred approach should be prioritised over standardisation. Patients were able to identify different activities or subject matter that they would be interested in. On visits to units where arts activities had been available, previous patients who had declined participation provided reasons for non-engagement. These conversations revealed that some patients wanted the opportunity to learn artistic skills, as opposed to contributing to a collaborative art piece that was outside their creative control. This contrasted sharply with consultations with some healthcare professionals, who reported many patients had limited capacity and would benefit more from a colouring exercise. The underpinning theoretical framework for the intervention was flow [34], discussed further below, which asserts in order to induce a flow state; the activity must be challenging enough to engage in a participant, but not too challenging to induce distress or anxiety [34]. Therefore, the contrast in feedback further reinforced the need for a more person-centred approach due to the clear variation in patient interests, confidence and skill level.

**Fig. 3:**
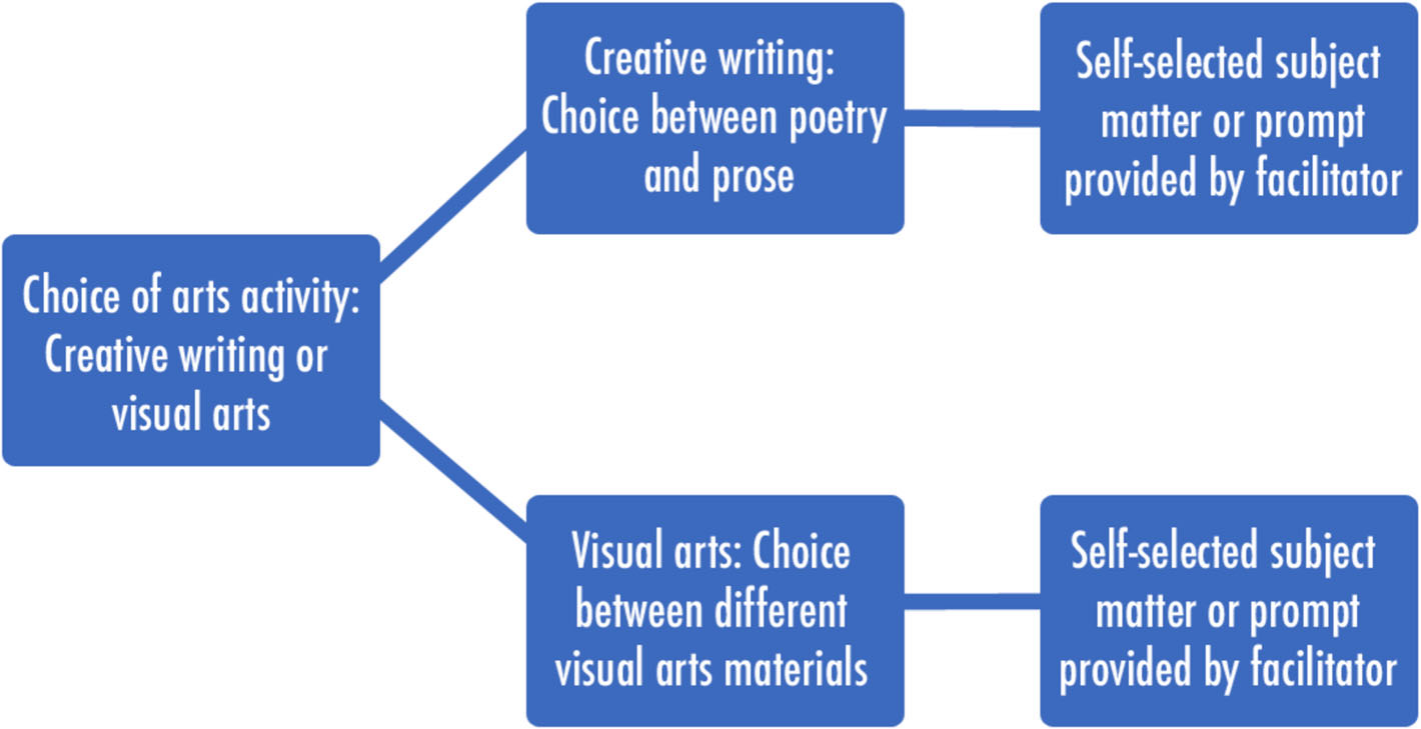
Overview of choices included in each facilitated art session

These issues were then discussed again with the IAG to develop a person-centred, flexible approach to an intervention that could be delivered within a structure that would allow replication. This resulted in the inclusion of a series of choices that participants made at the beginning of each art session. A brief overview of these choices is illustrated in the flow diagram in Fig. 3.

### Research-related projects

#### Scoping review

A total of 28 papers were identified for the scoping literature review, including articles focused on patients receiving haemodialysis (23), and patients with a cancer diagnosis (5). A total of 1585 patients and 24 members of healthcare professionals provided data across all studies. The arts-based interventions used included music listening interventions within haemodialysis units [35–45], art viewing interventions within bone marrow transplant units [46, 47], arts-in-medicine programmes within haemodialysis units [17, 18, 48–50], visual art-making interventions with bone marrow transplant units [51–53], a creative activity programme on a haemodialysis unit [54] and a participatory music programme within a haemodialysis unit [55].

The scoping review highlighted the existing evidence base for highly complex arts-based interventions focused on qualitative research and programme evaluations using non-validated questionnaires or surveys; therefore, none of the described interventions were developed for the purpose of rigorous evaluation. Components of the interventions identified from the review were discussed with the IAG, these included the length of time for each individual session, direct one-to-one facilitation during the session with a facilitator and key contextual issues relating to delivery. The included and excluded intervention components are outlined in Table 1.

**Table 1:** Included and excluded intervention components identified from the scoping review

Included intervention components	Reasons	Excluded intervention components	Reasons
One-to-one facilitation	Physical restrictions of patients (vascular access). Reduce burden on healthcare professionals	Art trolley/communal art supplies	Infection control concerns
Visual arts activities	Implementation likely to be feasible	Collaborative group projects	Person-centred approach required to induce flow
Creative writing activities	Implementation likely to be feasible	Display or spotlight ‘wall’	Initially planned, however, level of patient anxiety resulted in the removal of this component during the feasibility study
Flexibility of choice	To promote engagement and facilitate the inducement of a flow state.	Music activities	Existing empirical evidence base, including meta-analyses on efficacy.
Activity log and time limited sessions	To allow evaluation of fidelity and adherence within the framework of an RCT	Requirement for facilitator to hold a Bachelor of Arts.	Not well justified within the literature, and limited resources of the feasibility study would not enable recruitment of a professional artist.

#### Realist synthesis

The realist synthesis contributed to the development of a theoretical framework that included two phases of intervention delivery, firstly delivery of person-centred art activities during haemodialysis and secondly, display of completed artwork [16]. The interventions that were included in the review appeared to trigger a number of underlying mechanisms, including flow [34], a state of optimal engagement which is thought to contribute to an altered perception of time, specifically the sensation that time is passing quickly. After discussing candidate theories outlined in the realist synthesis with the IAG, flow was identified as the most appropriate theoretical framework to guide the intervention.

The concept of a ‘flow state’ resulted from Mihaly Csikszentmihalyi’s observations of artists at work [34]. Flow is a state of effortless enjoyment and optimal experience induced when a person undertakes a challenging task with clear goals, and has the skills required to meet the challenge [34]. The conditions required for inducement of a flow state include clarity of goals, immediate feedback, perceived challenges and skill development. To achieve a flow state, it is important the intervention presents a challenge to participants, provides them with an opportunity to learn and develop new skills, but is not too challenging that the activity is no longer manageable.

The synthesis also identified a number of contextual factors that act as barriers or facilitators to implementation. Implementation was hindered by constraints of the haemodialysis unit and patients’ lack of confidence in their artistic skills. In the literature, these issues were addressed through a flexible approach to implementation and support from healthcare professionals.

### Undertake abstract conceptualisation

This stage of the development process involves conceptualising the potential overview of the intervention and ultimately developing a concrete idea of how the intervention will look and be implemented in practice.

#### Generate ideas

A list of potential artistic activities, materials and delivery strategies were developed by the primary researcher (CC) and these were reviewed in relation to the available literature, the previously gathered information from consultations with patients and healthcare professionals, and the restrictions of the haemodialysis setting, by the IAG, alongside with individual members of the IAG over several months.

The clinical context of intervention delivery was the primary factor that limited the available artistic activities, as materials could only be included if they could be used by a person who was restricted to a hospital bed in an open, busy renal unit, whilst connected to a dialysis machine. This meant that the activities could not be overly disruptive, could not require substantial movement, could not require large equipment and could be delivered with minimal mess. Additionally, it was recommended that in order to effectively facilitate the intervention, the activities had to use materials that the primary researcher (CC) was comfortable and skilled in using themselves. Consequently, it was decided that creative writing and certain forms of visual arts were the most appropriate activities to offer within this setting.

The choice of specific materials and tools were also discussed collaboratively with the IAG. It was advised that the materials be as accessible as possible as some patients may have problems with dexterity. This led to the inclusion of several additional items, including pencil grips, drawing boards and drawing board clips. The issue of minimising potential mess of the materials was also explored, and a watercolour brush pen, as opposed to a brush, was included to ensure open containers of water were not necessary for delivery

#### Develop logic model

**Fig. 4:**
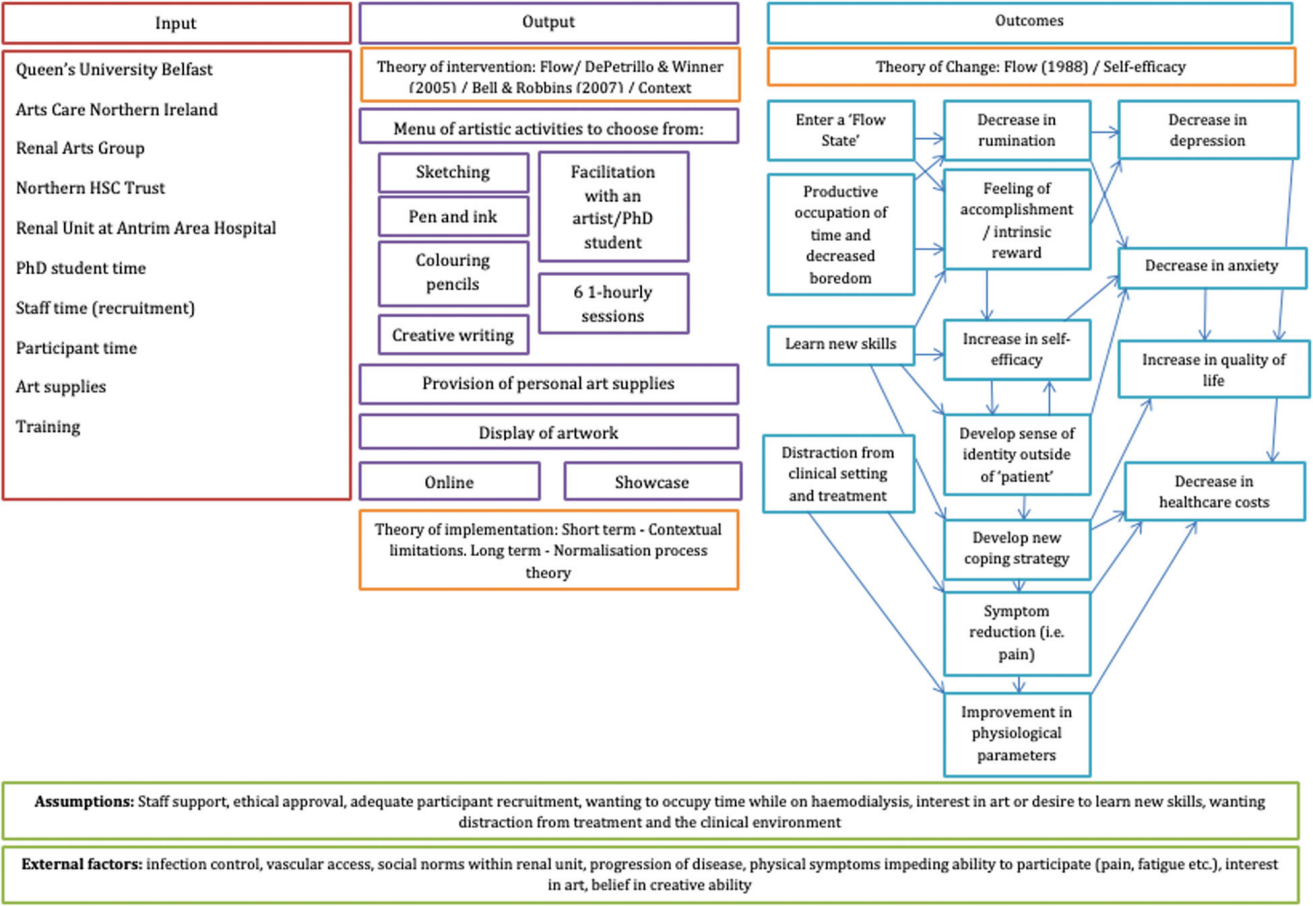
Logic model of the arts-based intervention developed prior to the feasibility study

The logic model in Fig. 4 illustrates an initial logic model developed prior to delivery to reflect the final product, reviewed and approved by all members of the IAG, that was created throughout the development process. The model outlines core inputs, outputs, anticipated outcomes, and potential underlying mechanisms that may contribute to improvement in the outcome measures used in the pilot cluster RCT.

### Final product—intervention for pilot cluster RCT

The final product of this process was a complex arts-based intervention for delivery within a pilot cluster RCT, to assess its acceptability within a future definitive trial. Therefore, it is anticipated that future modifications will be made to the intervention to improve delivery within a trial framework, and to scale-up implementation across multiple sites. A manualised version of this final product was drafted following delivery to promote transparency and replication, and is provided as supplementary material. This manual is based on the development process, the final product, data from the activity logs and experience of facilitation. The manual was revised and approved by the IAG.

#### Setting

The intervention setting was the haemodialysis unit during haemodialysis treatment. The sessions took place after all nursing procedures required to commence dialysis had taken place, thus minimising disruption to the clinical team. Prior to delivering the intervention, the facilitator introduced themselves to the clinical team, and explained the general content and purpose of the art sessions.

#### Facilitator

The intervention was facilitated by the primary researcher (CC) who is a registered mental health nurse with an A level in Art and Design. The facilitator felt comfortable using the arts materials, instructing participants on how to use them safely within the clinical environment and how to develop skills to improve their artistic abilities. The facilitator maintained an ongoing personal arts practice throughout the development and delivery of the intervention [25], which helped develop a compassionate and flexible approach to the arts. The facilitator also had strong communication skills that allowed the provision of constructive feedback whilst identifying and cultivating existing skillsets.

#### Materials

The following materials, informed and agreed by the IAG, were provided to each participant in their own personal arts pack: Sketch book, graphite pencils, graphic pens, watercolour paints, watercolour brush pen with in-built water container, colouring pencils, drawing board, drawing board clip, eraser, sharpener and pencil grip. Each participants’ materials were stored within a tote bag that was kept on site.

#### Art sessions

The sessions were flexible and person-centred to ensure optimal engagement with the creative process, an approach identified through consultations with patients and healthcare professionals, the scoping review, and confirmed by the IAG, and increase the chance of inducing a flow state, as highlighted in the realist synthesis. The sessions involved one-to-one facilitation which ensured accessibility as some participants found it difficult to use the materials whilst connected to a haemodialysis machine, and also assisted in the development of skills as the facilitator was able to observe patients and provide timely constructive feedback. The first session involved contextualising the intervention within the underpinning theory of flow by explaining each session was an opportunity to learn, experiment, practice and develop different skills, with an emphasis on experimentation and creating an enjoyable experience, in order to reduce anxiety about the quality of the final product. The display component of the intervention, that had been identified during the realist synthesis, was achieved during delivery by showing their completed works to interested patients and healthcare professionals with the patient’s permission. This less formal approach to display was utilised after discussions with the IAG, following recruitment to the feasibility study [20], as the level of anxiety amongst patients resulted in a reluctance to exhibit their completed works publicly.

#### Choice of artistic activities

The choice of artistic activities was identified through the scoping reviews, personal arts practice, consultation with patients and healthcare professionals, and were confirmed through the IAG. Patients were given a choice between either visual art activities or creative writing; however, this initial choice was not final, instead the patient was aware they could change their mind within or between sessions. As some patients had not engaged with creative writing or visual arts since they attended school, it took time and experimentation to help them identify their preferred activity.

#### Creative writing

When a patient identified creative writing as their preferred activity, they were provided with a choice between poetry and short stories. The facilitator was ready to provide a variety of different writing prompts for participants who found it difficult to identify subject matter. The writing process was at times highly collaborative, with the facilitator working with the patient to develop their poem or story by providing immediate feedback.

#### Visual art

Participants who chose visual arts activities had a variety of activities to choose from, including sketching with graphite pencils, sketching with colouring pencils, watercolour painting and graphic pen sketching. Some patients were highly anxious about their perceived lack of ability, in which case, the facilitator suggested watercolour painting or colouring pencil sketching over an image that was already drawn by the facilitator. Some participants found it difficult to identify subject matter, in which case the facilitator provided ideas and prompts in the form of reference images. Whilst the first session was focused on familiarising the individual with the arts materials and how to use them, the following sessions were focused on developing skills specific to the materials being used.

### Assessment of dose, adherence and fidelity

#### Intervention duration

Assessment of dose in complex intervention research requires a level of standardisation across the amount of intervention being administered to ensure that generalisations and comparisons can be made across participants, and any associations between dose and response can be explored during evaluation [56]. The scoping review highlighted that dose is not considered or reported in the literature that describes the provision of arts-in-medicine programmes for patients receiving haemodialysis. To allow assessment of dose across participants, the intervention was time-limited, both in terms of the amount of sessions and session length. Each participant was provided 6 sessions in total, a dose that was determined through consultations with key stakeholders. Each session was limited to 1 h, a time frame that was identified from the portion of the scoping review related to arts-based interventions for patients with cancer. The amount of sessions and length of sessions were reviewed and approved by the IAG. The sessions took place twice a week over a period of 3 weeks. This was to facilitate flexibility in the implementation, as patients typically attend haemodialysis 3 days a week. The IAG identified this important component of implementation, as it ensured that if a patient was unable to engage with a session 1 day they were provided the opportunity to reschedule for another day in the week.

#### Record of sessions

To allow the assessment of fidelity of delivery and adherence to the intervention, an activity log was completed by the facilitator following each session. Activity logs were identified from the scoping review as they have been described in previous research relating to arts-in-medicine programmes; however, details of these logs were not provided and the logs were utilised as a method of communication between facilitators as opposed to capturing information for empirical research [48]. The activity log outlined the date and time of the shift when the session took place, the activities undertaken with a list of all items used in the session, and the amount of time the participant was engaged in each session. Reasons for non-participation were recorded, for example, if a patient was too fatigued to engage during one shift and asked to reschedule to later in the week. Pertinent comments made by patients or healthcare professionals that related to the experience of the intervention were also captured. The description of activities included not only the artistic medium and subject matter but also practical considerations to support the patient during the session, reference images used by participants and any plans for the subsequent session.

## Discussion

This paper is the first to describe the development process of a replicable and person-centred complex arts-based intervention within a haemodialysis setting. Whilst the intervention is placed within a larger feasibility study where issues surrounding adherence, dose, fidelity and acceptability are explored [20], this article provides a worked example of a collaborative combination approach to the development of a complex arts-based intervention.

As the intervention was developed for the purpose of evaluation within a trial framework, the approach to development had to balance key considerations necessary for implementing a person-centred complex arts-based intervention with the restrictions necessary for assessment within a trial, for example capturing information relating to duration of engagement, a set number of sessions, standardised provision of materials and a pre-selected framework of options. Arts in health research has been consistently criticised for the dearth of RCTs evaluating complex arts-based interventions [57]. The gap in the evidence could be a consequence of the tension between the person-centred approach of the arts and the structure of trial methodology. It is important, for example, that when considering fidelity of the intervention in a cluster RCT that participants should receive the same core, active components across all sites. There is additional debate as to whether it is appropriate to instrumentalise the arts and subject them to scientific standards, due to the perception that art and science are disparate fields with conflicting priorities [58]. However, the potential for arts-based interventions to address unmet needs and improve the quality of life for people with limited access outside of the healthcare context, should arguably be a higher priority [57]. Therefore a pragmatic approach was taken with the development of this intervention in an attempt to balance flexibility and standardisation.

A significant strength of the development process was the utilisation of INVOLVE’s consultation and collaboration approach [23]. This ensured the development process continuously placed the priorities of patients, healthcare professionals and other key stakeholders as the focus of the intervention. Additionally, it ensured that factors likely to contribute to the acceptability of the intervention were identified prior to delivery, due to the unique insight patients with lived experience provided during the development process. An additional strength was the utilisation of the Arts in Health development framework [22], as the inherent flexibility of the framework allowed an iterative approach to development whilst also ensuring the final product was grounded in the existing evidence base and was tailored to the unique experience of the target group.

The scope of this article limits the generalisability of the findings to the immediate context of delivery within the larger study. This overview of the development process does not provide insight into the acceptability or fidelity of the described arts-based intervention. Therefore, scaling up of the intervention for a larger RCT or general implementation in practice may require modification to some aspects of delivery [56]. For example, the intervention outlined in this paper was delivered by a single person who was not a trained artist but was a qualified mental health nurse. Whilst their role delivering the intervention was distinct to their previous nurse training, it likely impacted their comfort in the clinical environment and their communication with participants. Therefore, when scaling up the intervention, this needs to be considered in relation to fidelity and the level of skill, experience and training the facilitators will need during implementation across multiple sites with multiple facilitators.

The development process also did not involve primary data collection with patients or healthcare professionals, as there is an existing evidence base that captures patients’ experiences of haemodialysis treatment and evaluations of arts-in-medicine programmes. Instead, key stakeholders were active contributors to the development process, and were essential to not only providing information but also synthesising and interpreting information and creating the final product.

There is a significant gap in the arts in health literature outlining the development processes for arts-based interventions, and future research should provide transparent overviews of development processes to further enhance the evidence base. Research into complex arts-based interventions should also explore implementation within different clinical settings to better understand the unique complexities associated with providing arts-based interventions across different patient groups and clinical environments.

## Conclusion

In patients receiving haemodialysis, there is a dearth of evidence on the effectiveness of complex arts-based interventions for improving health, well-being and quality of life, and consequently this can limit access through constraints on funding and resources. This paper provided a worked example of developing a complex arts-based intervention for patients receiving haemodialysis using the Arts in Health development framework, demonstrating that it is possible to develop these intervention in way that enables delivery within a trial framework. Establishing a robust evidence base for arts-based interventions that have been developed in collaboration with patients, healthcare professionals and key stakeholders, can help promote access to highly acceptable, safe and holistic care.

## Supplementary Information

**Additional file 1.** : GUIDED – a guideline for reporting for intervention development studies. Blank Checklist. Intervention manual supplemental material

## Data Availability

No primary data was collected; however, the intervention manual attached as supplementary material.

## Abbreviations

AVF: Arteriovenous fistula
CEO: Chief executive officer
ESKD: End-stage kidney disease
IAG: Interdisciplinary advisory group
NHS: National Health Service
NIHR: National Institute for Health Research
RCT: Randomised controlled trial
RRT: Renal replacement therapy

## References

[1] Carswell C, Reid J, Walsh I, Johnston W, McAneney H, Mullan R (2020). A mixed-methods feasibility study of an arts-based intervention for patients receiving maintenance haemodialysis. BMC Nephrol.

[2] Registry UR: UK Renal Registry 22nd Annual Report - data to 31/12/2018. In. Bristol, UK; 2020.

[3] Haugen CE, Chu NM, Ying H, Warsame F, Holscher CM, Desai NM (2019). Frailty and access to kidney transplantation. Clin J Am Soc Nephrol.

[4] van der Willik EM, Hemmelder MH, Bart HA, van Ittersum FJ, Hoogendijk-van den Akker JM, Bos WJW, Dekker FW, Meuleman Y: Routinely measuring symptom burden and health-related quality of life in dialysis patients: first results from the Dutch registry of patient-reported outcome measures. Clinical Kidney Journal 2020. 10.1093/ckj/sfz192 PMC8286800 34285801

[5] Bublitz L, Hiremagalur B, Purtell L, Sowa M, Gillespie K, Bonner A: 33. Tracking patient-reported symptoms in people undergoing dialysis: the LEOPARD study. Renal Society of Australasia Journal 2018, 14:29-29.

[6] Lindsay H, MacGregor C, Fry M (2014). The experience of living with chronic illness for the haemodialysis patient: an interpretative phenomenological analysis. Health Sociol Rev.

[7] Shaw R (2015). Being-in-dialysis: the experience of the machine–body for home dialysis users. Health.

[8] Herlin C, Wann-Hansson C (2010). The experience of being 30–45 years of age and depending on haemodialysis treatment: a phenomenological study. Scand J Caring Sci.

[9] Gullick J, Monaro S, Stewart G (2017). Compartmentalising time and space: a phenomenological interpretation of the temporal experience of commencing haemodialysis. J Clin Nurs.

[10] Moran A, Scott P, Darbyshire P (2009). Existential boredom: the experience of living on haemodialysis therapy. Med Humanities.

[11] Palmer S, Vecchio M, Craig JC, Tonelli M, Johnson DW, Nicolucci A (2013). Prevalence of depression in chronic kidney disease: systematic review and meta-analysis of observational studies. Kidney Int.

[12] Semaan V, Noureddine S, Farhood L: Prevalence of depression and anxiety in end-stage renal disease: a survey of patients undergoing hemodialysis. (1532-8201 (Electronic)); 2018. 10.1016/j.apnr.2018.07.009 30220369

[13] Kokoszka A, Leszczyńska K, Radzio R, Daniewska D, Łukasiewicz A, Orzechowski WM (2016). Prevalence of depressive and anxiety disorders in dialysis patients with chronic kidney disease. Arch Psychiatry Psychother.

[14] Ossareh S, Tabrizian S, Fau - Zebarjadi M, Zebarjadi M Fau - Joodat RS, Joodat RS: Prevalence of depression in maintenance hemodialysis patients and its correlation with adherence to medications. (1735-8604 (Electronic)); 2014. 25362222

[15] Chilcot J, Guirguis A, Friedli K, Almond M, Day C, Da Silva-Gane M (2017). Depression symptoms in haemodialysis patients predict all-cause mortality but not kidney transplantation: a cause-specific outcome analysis. Ann Behav Med.

[16] Carswell C, Reid J, Walsh I, McAneney H, Lee JB, Noble H. Complex arts-based interventions for patients receiving haemodialysis: a realist review. Arts Health. 2020:1–27. 10.1080/17533015.2020.1744173. 10.1080/17533015.2020.1744173 32233723

[17] Moss H: Punchestown kidney research fund progress report: art programme in the dialysis unit 2011-2012. In. Dublin; 2012.

[18] Rowe N, Jones CH, Seeger L, Greaves G, Holman C, Turner H (2011). Forgetting the machine: patients’ experiences of engaging in artwork while on renal dialysis. J Applied Arts Health.

[19] Ogden J (2017). QALYs and their role in the NICE decision-making process. Prescriber.

[20] Carswell C, Reid J, Walsh I, McAneney H, Noble H (2019). Implementing an arts-based intervention for patients with end-stage kidney disease whilst receiving haemodialysis: a feasibility study protocol. Pilot Feasibility Studies.

[21] O’Cathain A, Croot L, Sworn K, Duncan E, Rousseau N, Turner K (2019). Taxonomy of approaches to developing interventions to improve health: a systematic methods overview. Pilot Feasibility Studies.

[22] Fancourt D: Arts in health: designing and researching interventions: Oxford University Press; 2017.

[23] Approaches to public involvement in research [https://www.invo.org.uk/posttyperesource/approaches-to-public-involvement/]. Accessed 25 Feb 2019.

[24] Carswell C, Reid J, Walsh I, Noble H (2018). Arts-based interventions for hospitalised patients with cancer: a systematic literature review. Br J Healthc Manag.

[25] Carswell C (2020). Claire Carswell: visceral and aesthetic imagery. J Visual Comm Medicine.

[26] W K Kellogg Foundation. Logic Model Development Guide: Using Logic Models to Bring Together Planning, Evaluation, and Action, Logic Model Development Guide. Battle Creek; 2004.

[27] Connolly P, Biggart A, Miller S, O’Hare L, Thurston A: Using randomised controlled trials in education: Sage; 2017, DOI: 10.4135/9781473920385.

[28] Basu R (2004). Tools for analysis-PESTLE analysis in implementing quality: a practical guide to tools and techniques.

[29] All-Party Parliamentary Group on Arts H, Wellbeing: Creative health: the arts for health and wellbeing: All-Party Parliamentary Group on Arts, Health and Wellbeing; 2017.

[30] Sonke J, Lee J, Rollins J, Carytsas F, Helgemo M, Imus S (2017). Talking about arts in health: a white paper addressing the language used to describe the discipline from a higher education perspective.

[31] Galway University Hospitals Arts Trust. The Magician and the Swallow’s Tale, Artsandhealth.ie.; 2013. Available at: http://www.artsandhealth.ie/case-studies/the-magician-and-the-swallows-tale/. Accessed 25 Feb 2019.

[32] Kidney Care UK. Haemodialysis (HD) | Kidney Care UK; 2021. Available at: https://www.kidneycareuk.org/about-kidneyhealth/treatments/dialysis/haemodialysis/. Accessed 12 June 2021.

[33] Chiaranai C (2016). The lived experience of patients receiving hemodialysis treatment for end-stage renal disease: a qualitative study. J Nurs Res.

[34] Csikszentmihalyi M, Csikzentmihaly M: Flow: the psychology of optimal experience, vol. 1990: Harper & Row New York; 1990.

[35] Schuster BL (1985). The effect of music listening on blood pressure fluctuations in adult hemodialysis patients. J Music Ther.

[36] Pothoulaki M, Macdonald RA, Flowers P, Stamataki E, Filiopoulos V, Stamatiadis D (2008). An investigation of the effects of music on anxiety and pain perception in patients undergoing haemodialysis treatment. J Health Psychol.

[37] Cantekin I, Tan M (2013). The influence of music therapy on perceived stressors and anxiety levels of hemodialysis patients. Ren Fail.

[38] Lin Y-J, Lu K-C, Chen C-M, Chang C-C (2012). The effects of music as therapy on the overall well-being of elderly patients on maintenance hemodialysis. Biological Res Nursing.

[39] Koca Kutlu A, Eren AG (2014). Effects of music on complications during hemodialysis for chronic renal failure patients. Hemodial Int.

[40] Midilli TS, Ergin E, Yılmaz H (2017). The effects of listening to music on vital signs and anxiety in hemodialysis patients. Int J Health Sci Res.

[41] Hou Y-C, Lin Y-J, Lu K-C, Chiang H-S, Chang C-C, Yang L-K (2017). Music therapy-induced changes in salivary cortisol level are predictive of cardiovascular mortality in patients under maintenance hemodialysis. Ther Clin Risk Manag.

[42] Shabandokht-Zarmi H, Bagheri-Nesami M, Shorofi SA, Mousavinasab SN (2017). The effect of self-selected soothing music on fistula puncture-related pain in hemodialysis patients. Complement Ther Clin Pract.

[43] Momennasab M, Ranjbar M, Najafi SS (2018). Comparing the effect of listening to music during hemodialysis and at bedtime on sleep quality of hemodialysis patients: a randomized clinical trial. Eur J Integrative Med.

[44] Melo GAA, Rodrigues AB, Firmeza MA, ASdM G, PPd O, Caetano JÁ. Musical intervention on anxiety and vital parameters of chronic renal patients: a randomized clinical trial. Revista latino-americana de enfermagem. 2018;26(0). 10.1590/1518-8345.2123.2978. 10.1590/1518-8345.2123.2978 PMC5863277 29538579

[45] Kishida M, Yamada Y, Inayama E, Kitamura M, Nishino T, Ota K (2019). Effectiveness of music therapy for alleviating pain during haemodialysis access cannulation for patients undergoing haemodialysis: a multi-facility, single-blind, randomised controlled trial. Trials.

[46] Hanson H, Schroeter K, Hanson A, Asmus K, Grossman A: Preferences for photographic art among hospitalized patients with cancer. In: Oncology Nursing Forum: 2013: Oncology Nursing Society; 2013: E337-E345. 10.1188/13.ONF.E337-E345 23803278

[47] McCabe C, Roche D, Hegarty F, McCann S (2013). ‘Open window’: a randomized trial of the effect of new media art using a virtual window on quality of life in patients' experiencing stem cell transplantation. Psycho-Oncology.

[48] Ross EA, Hollen TL, Fitzgerald BM (2006). Observational study of an arts-in-medicine program in an outpatient hemodialysis unit. Am J Kidney Dis.

[49] Field G: The benefit of therapeutic art sessions for patients in a renal dialysis unit: a guide to establishing art in acute hospital renal dialysis services. In*.* Dublin; 2007.

[50] Corrigan C, Peterson L, McVeigh C, Lavin P, Melotte G, Wall C (2017). The perception of art among patients and staff on a renal dialysis unit.

[51] Lawson LM, Glennon C, Amos M, Newberry T, Pearce J, Salzman S (2012). Patient perceptions of an art-making experience in an outpatient blood and marrow transplant clinic. Eur J Cancer Care.

[52] Lawson M, Cline J, French A, Ismael N. Patient perceptions of a 1-h art-making experience during blood and marrow transplant treatment. Eur J Cancer Care. 2017;26(5). 10.1111/ecc.12482. 10.1111/ecc.12482 27195450

[53] Lawson LM, Williams P, Glennon C, Carithers K, Schnabel E, Andrejack A, Wright N: Effect of art making on cancer-related symptoms of blood and marrow transplantation recipients. In: Oncology Nursing Forum*:* 2012; 2012. 10.1188/12.ONF.E353-E360 22750906

[54] Pedreira-Robles G, Vasco-Gómez A, Martínez-Delgado Y, Herrera-Morales C, Junyent-Iglesias E, Ho-Wong TM (2018). Using creative activities to improve treatment perceptions of patients on hemodialysis. Nephrol Nurs J.

[55] Grehan M: Waterford healing arts trust evaluation of music in renal dialysis programme 2009. In. Waterford: Waterford Healing Arts Trust; 2010.

[56] Moore GF, Audrey S, Barker M, Bond L, Bonell C, Hardeman W (2015). Process evaluation of complex interventions: Medical Research Council guidance. BMJ.

[57] Fancourt D, Finn S (2019). What is the evidence on the role of the arts in improving health and well-being?.

[58] Phillips K (2019). A constructive-critical response to creative health: the arts for health and wellbeing (July 2017) by the all–party parliamentary group on arts, health and wellbeing. Int J Art Therapy.

