# Supplementary material for: Development of a complex arts-based intervention for patients with end-stage kidney disease whilst receiving haemodialysis
Source: Pilot Feasibility Stud. 2021 Jun 16;7:127. doi: 10.1186/s40814-021-00868-2 (PMC8207758; doi:10.1186/s40814-021-00868-2)
Supplement: Supplementary file 1 — Additional file 1. : GUIDED – a guideline for reporting for intervention development studies. Blank Checklist. Intervention manual supplemental material [file 40814_2021_868_MOESM1_ESM.docx]

# Intervention manual

## Intervention theory and aim

The primary theory underpinning this arts-based intervention for patients receiving haemodialysis is the positive psychological concept of ‘Flow’ (Csikszentmihalyi, 1990). The concept of a ‘Flow State’ resulted from Mihaly Csikszentmihalyi’s observations of artists at work. Flow is a state of effortless enjoyment and optimal experience induced when a person undertakes a challenging task with clear goals, and has the skills required to meet the challenge (Csikszentmihalyi, 1990).

The conditions required for inducement of a Flow State include:

- Clarity of goals
- Immediate feedback
- Perceived challenges
- Skill development

To achieve a flow state, it is important the intervention presents some degree of challenge to participants, provides them with an opportunity to learn and develop new skills, but is not too challenging that the activity is no longer manageable. Figure 5 illustrates the intervention in an expanded logic model, developed following implementation of the intervention, that provides an overview of the specific contextual considerations and suggestions for facilitation that promoted engagement during implementation.

## Intervention Facilitator

The intervention was facilitated during the feasibility study by a PhD student who was also a registered mental health nurse with an A level in Art and Design. It is recommended that the facilitator feels comfortable using the arts materials, instructing participants on how to use them safely within the clinical environment and how to develop skills to improve their artistic abilities. While a qualified artist with experience in healthcare settings would be best placed to deliver the intervention due to their familiarity with the setting and the artistic activities, the feasibility study demonstrated that higher arts education or professional training in the arts is not necessary for sustained engagement from participants. However, it is essential that the facilitator feels competent and confident engaging in the arts activities themselves, in order to be provide meaningful feedback and guidance to patients, demonstrate techniques related to the activities, and provide outlines for participants who are less confident in their drawing abilities.

The facilitator must be competent in using the materials and activities, and able to reassure participants on the process of skill development. An ongoing personal arts practice can assist the facilitator in developing ideas, and acquainting themselves with different techniques and materials (Carswell, 2019). The personal characteristics of the facilitator are important, and should enable a compassionate and flexible approach to the arts, whilst they must also have strong communication skills that allow the provision of constructive feedback whilst identifying and cultivating existing skillsets.

## Context of intervention

### Setting

- The intervention setting is the haemodialysis unit during the patient’s haemodialysis treatment. The session should take place after all nursing procedures required to commence dialysis has taken place, thus minimise disruption to the clinical team.
- The clinical team should be aware that the sessions are taking place and what the sessions will involve. Prior to implementation the artist should introduce themselves to the clinical team, explain the general content and purpose of the art sessions, and explain that the intention is to be as undisruptive as possible. Therefore, if members of the clinical team need access to the patient they should feel comfortable interrupting the sessions to provide clinical care.

### Intervention duration

- Each participant should be provided 6 sessions in total. Each session should be approximately an hour, although if a patient completes their work or requests the session ends prior to an hour it can end sooner.
- The sessions will take place twice a week over a period of three weeks. This is to facilitate flexibility in the implementation, as patients typically attend haemodialysis 3 days a week. If a person is unable to engage with a session one day they should be provided the opportunity to reschedule for another day in the week.
- Following the completion of each session the artist should complete an activity log, this will detail the activities completed during the session, ideas for future sessions and the length of time the session lasted. This log can be printed and kept with the facilitator (in the case of research it can be stored in the trial management file on site), or can be completed digitally on an encrypted computer depending on the resources available.

### Materials

It is suggested that the following materials are provided to each participant in their own personal arts pack:

- Sketch book
- Graphite pencils
- Graphic pens
- Watercolour paints
- Watercolour brush pen with in-built water container
- Colouring pencils
- Drawing board
- Drawing board clip
- Eraser
- Sharpener
- Pencil grip

These materials will be stored within a tote bag that will be kept on site to reduce the risk that the materials may be lost or forgotten if brought home by patients. The intervention facilitator themselves should also bring in their own sketchbook and pencils to demonstrate techniques and provide reference images. These materials should be able to be wiped down between patients to reduce any risk of cross-contamination.

## The arts-based intervention

This section is intended to provide an overview of the basic process of each session. The sessions should be flexible and person-centred to ensure optimal engagement with the creative process and increase the chance of inducing a flow state. The sessions involve one to one facilitation with an artist to ensure accessibility as some participants may find it difficult to use the materials whilst connected to a haemodialysis machine, but also to assist in the development of skills as the artist will be able to observe the patient during the sessions and be able to provide genuine constructive feedback. It is important that the artist also presents the activities as having a skill development focus, with an emphasis on experimentation and creating an enjoyable experience, in order to reduce anxiety about the quality of the final product. The first session should involve contextualising the intervention within the concept of Flow by explaining to the participant that each session is an opportunity to learn, experiment, practice and develop different skills. The arts should be framed as a skill with potential for development as opposed to an innate talent, and therefore the emphasis should be on the learning and development process as opposed to the final result. If a participant has limited prior experience with art they should be reassured that they while they may find it challenging, they will improve over time as they learn different techniques.

While this framework is not intended to be prescriptive, as ideally the sessions should be patient led, it provides an overview of different choices the participants can make throughout the sessions.

### Choice of artistic activities

Patients should be given a choice between either visual art activities or creative writing, however this initial choice should not be viewed as final; instead the patient should be made aware that they can change their mind within or between sessions. As some patients may not have engaged with creative writing or visual arts since they attended school it may take some time and experimentation to help them identify their preferred activity. The artist can also use their own assessment of the individual to suggest different activities that they feel would be of most benefit, for example, a person with limited use of their hands may benefit from creative writing in an adapted story-telling form as opposed to visual arts.

#### Creative writing

If a patient identifies creative writing as their preferred activity they will also be provided with a choice between poetry and short stories. The patient themselves may have an idea of their preferred subject matter, however it is likely that those with limited experience may find identifying subject matter difficult, especially within the first few sessions. Therefore, the artist should be ready to provide a variety of different writing prompts. These could include prompts relating to:

- Seasons
- Holidays
- Family
- Food
- History
- Music
- TV, films and books
- Nature

This list is not comprehensive but is intended to give an overview of very general subject areas that can be used to develop more specific prompts or assist the patient in focusing the scope of their writing. Writing exercises and writing prompts are also readily available online if the patient requires more direction initially.

The writing process can be highly collaborative with the artist actively working with the patient to develop their poem or story by providing immediate feedback, praise and/or offering suggestions where appropriate. It can also be helpful for the artist to work on their own piece of writing to reduce potential feelings of self-consciousness the patient may experience when sharing their work. Certain pieces of creative writing may require multiple sessions to complete, such as developing and writing a short story, so it is important to remind the participant that they have time to work on the piece and it does not need to be completed within a single session.

#### Visual art

Participants who choose visual arts activities will have a variety of activities to choose from, including sketching with graphite pencils, sketching with colouring pencils, watercolour painting and graphic pen sketching. Some patients may be highly anxious about their perceived lack of ability to draw, in which case the artist will suggest watercolour painting or colouring pencil sketching over an image that can be drawn by the artist themselves. Similar to creative writing, some participants may find it difficult to identify subject matter for their visual art, in which case the artist should provide a variety of different ideas. These ideas should ideally be presented in the form of reference images to assist the person during the session. During initial sessions it may be best to suggest simpler images, depending on the person’s previous experience with the arts materials.

While the first session should focus on familiarising the individual with the arts materials and how to use them, the following sessions should focus on developing skills specific to the materials being used. The following techniques and principles can be explored depending on the facilitator and individual’s skill level, the activity and the artistic materials they are using:

- Drawing: Deconstructing image into basic shapes, artistic principle of ‘draw what you see’, shading and light.
- Graphite pencils: Smooth shading varying pressure, hatching, and contour.
- Colouring pencils: Smooth shading varying pressure, hatching, and contour.
- Watercolours: Water control and transparency, gradients and blending.
- Pen and ink: Hatching: linear hatching, cross hatching, contoured hatching, pointillism, line and wash.

### General guidelines

- At the end of each session the subsequent session should be planned by the artist and patient. This not only maintains the patient’s motivation but ensures the artist will be able to prepare the appropriate reference images or prompts.
- Timely, consistent feedback is required in order to induce a flow state. Throughout each session the artist should be observing or collaborating with the patient and providing suggestions, praise and reassurance throughout.
- Review of previously completed work can highlight the development of skills. As the sessions progress the artist should use time within each session to reflect on previously completed work and draw direct comparison to illustrate improvement.
- As sessions progress the complexity of activities should increase in order to challenge the participant as they become comfortable with the different activities and techniques. However, this should also be patient led and extra time can be spent.
- The artist should encourage patients to identify subjects that are of interest to them. This may be difficult initially however while prompts may help participants familiarise themselves with the activities and materials, combining them with subjects of personal interest can further enhance engagement.
- The artist should maintain awareness of the clinical environment and infection control issues. The artist should wash their hands in between patients, should be bare below the elbows and tie back any long hair. If there are any clinical concerns the artist should raise them with the nursing staff.
- The facilitator should be aware that there will likely be alarms going off in the immediate clinical environment. If the haemodialysis machine of the participant alarms the facilitator should be prepared to step aside while the nursing staff attend to the cause of the alarm. In some case this may only require a couple of seconds, whereas in other scenarios the facilitator may need to leave and return to the participant at a later time or date.
- Patients receiving haemodialysis may experience difficult symptoms such as pain, itchiness and fatigue. Some patients may feel too fatigued to engage, however others may welcome to opportunity to engage in an activity that will help them remain awake during their haemodialysis session, alternatively patients may find that the activities distract them from difficult symptoms such as itchiness or pain. Therefore, it is important that the facilitator is guided by the patient on whether these issues will impact their ability to participate.
- If the patient is comfortable and consents, feedback can also be provided by the healthcare professionals and other patients during the sessions. This can reinforce the feedback provided by the artist and build social capital amongst patients and staff.
